# 

**Published:** 1898-10-15

**Authors:** 


					CADBURY'S O W CADBUKY S
COOOA Fl M COCOA
ABSOLUTELY PURE. W^? ^ NO DRUGS OR ALKALIES USED.
% 629. Vol. XXY. [*gjgggg] Saturday,
Oct. 15th, 1898.
[Snb80sefp.nIerma,J PRICE TWOPENCE.

				

